# Strategies to Increase the Phosphorus Content in the Soil Profile of Vineyards Grown in Subtropical Climates

**DOI:** 10.3390/plants13172434

**Published:** 2024-08-31

**Authors:** Adriele Tassinari, Lincon Stefanello, Jean Michel Moura-Bueno, Gustavo Nogara de Siqueira, Guilherme Zanon Peripolli, Bianca Goularte Dias, Douglas Luiz Grando, William Natale, Carlos Alberto Ceretta, Gustavo Brunetto

**Affiliations:** 1 Department of Soils, Federal University of Santa Maria (UFSM), 1000 Roraima Avenue, Santa Maria 97105-900, RS, Brazil; tassinaridrica@gmail.com (A.T.); buenojean1@gmail.com (J.M.M.-B.); gustavo.nogara@acad.ufsm.br (G.N.d.S.); guiga.peripolli@gmail.com (G.Z.P.); goulartediasbianca@gmail.com (B.G.D.); douglas.agn@hotmail.com (D.L.G.); william.natale@ufsm.br (W.N.); carlosceretta@ufsm.br (C.A.C.); 2 Department of Agronomy, Federal Technological University of Paraná (UTFPR), Cerejeira Street, Santa Helena 85892-000, PR, Brazil; lincono@utfpr.edu.br

**Keywords:** phosphate fertilization, fertilizer application modes, subsoiling, grape must composition, subtropical viticulture

## Abstract

Phosphate fertilizers are applied to the soil surface, especially in vineyards in production in subtropical regions. Nowadays, phosphorus (P) is not incorporated into the soil to avoid mechanical damage to the root system in orchards. However, over the years, successive surface P applications can increase the P content only in the topsoil, maintaining low P levels in the subsurface, which can reduce its use by grapevines. For this reason, there is a need to propose strategies to increase the P content in the soil profile of established orchards. The study aimed to evaluate the effect of management strategies to (i) increase the P content in the soil profile; (ii) enhance the grape production; and (iii) maintain the grape must composition. An experiment on the ‘Pinot Noir’ grape in full production was carried out over three crop seasons. The treatments were without P application (C), P on the soil surface without incorporation (SP), P incorporated at 20 cm (IP20), P incorporated at 40 cm (IP40), and twice the P dose incorporated at 40 cm (2IP40). The P concentration in leaves at flowering and *veraison*, P content in the soil, grape production and its components, and chemical parameters of the grape must (total soluble solids, total polyphenols, total titratable acidity, total anthocyanins, and pH) were evaluated. The P concentration in leaves did not differ among the P application modes. The application of P associated with soil mobilization, especially at 20 cm depth, increased grape production. The P application modes did not affect the values of the chemical parameters of the grape must except for the total anthocyanins, which had the highest values when the vines were subjected to 2IP40. Finally, the P application and incorporation into the soil profile was an efficient strategy for increasing the grape production in full production vineyards.

## 1. Introduction

The subtropical soils associated with the grape production system are acidic and deficient in phosphorus (P), making it essential to characterize them through chemical analysis of the soil and leaves. When necessary, limestone should be applied to the soil surface and incorporated into the 0–20 cm layer and, if conditions allow, up to 30 or 40 cm deep before planting the grapevines [1,2]. However, we postulate that when the phosphate fertilizers applied in the pre-planting fertilization of grapevines are not incorporated into the deeper layers of the soil, the increase in P content at depth will be small, which can reduce the growth of the root system and the absorption of water and nutrients. If this happens, the nutritional status will be negatively affected, and a reduction in grape productivity and changes in the berries and grape must composition [3] are expected. 

Considering all of these, it is necessary to use strategies to increase the P content in the soil profile in vineyards under production, especially in subtropical and tropical soils with a high P adsorption capacity [4] where the incorporation of phosphate fertilizer may not have been carried out efficiently at depth in the pre-planting fertilization. One possibility in vineyards under production would be to apply the phosphate fertilizer to the surface of the soil and mobilize the soil up to 20 cm or, if possible, in deeper layers such as 40 cm using a subsoiler. However, there are scarce medium or long-term field experiments in subtropical climates, which have evaluated the effect of incorporation methods of the phosphate fertilizers for enhancing the phosphorus efficiency by grapevines in production, especially due to the possibility of root damage. However, the dynamics of the root system itself are little known. Assuming that the greatest intensity of roots is found in the region of the canopy projection, where fertilizers are normally applied, incorporation between the rows may enhance the growth of the root system and contribute to greater P absorption. Thus, the application of phosphate fertilizers associated with soil mobilization can also be a strategy in vineyards in production in order to avoid excess P in the surface layers of vineyards due to constant surface applications. This is because even in sandy soils, P applied to the surface is adsorbed 1:1 to clay minerals or Fe, Mn and Al oxides, which reduces its availability and migration in the profile [4,5], and it is predisposed to greater losses with increasing terrain slope [6]. 

Fruit crops are usually grown for several years, as is the case with grapevines, which can have production for up to 50 years. Normally, mineral fertilizers in vineyards are not incorporated so as not to damage the root system. However, when this happens, only adequate fertility is observed on the surface, but there are low concentrations of nutrients at depth, which tends to prevent the vine from expressing its productive potential. Therefore, the hypothesis of our study is that incorporating P between the rows of vineyards can contribute to greater absorption by the vines, given that applications normally take place on the surface and in the planting row. The study aimed to evaluate the effect of management strategies for (i) increasing the P content in the soil profile; (ii) enhancing the grape production; and (iii) maintaining the grape must composition.

## 2. Results

**Figure 1:**
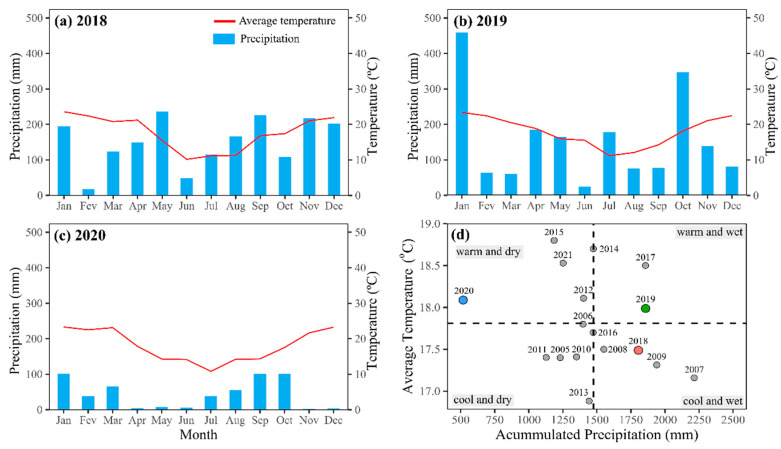
Average temperature (°C) and accumulated monthly precipitation (mm) in the years corresponding to the 2018/19 (**a**), 2019/20 (**b**), and 2020/21 (**c**) crop seasons; and average temperature and accumulated monthly precipitation recorded over the previous 17 years in the region where the study was carried out, Santana do Livramento, southern Brazil (**d**). The dashed lines represent the average temperature and average precipitation recorded for the entire period.

The highest precipitation (around 450 mm) in the 2018/19 crop season was observed in January when the grapes were ripening and being harvested. In the 2019/20 crop season, the highest precipitation was observed in October, coinciding with the end of budbreak and the start of flowering. In the 2020/21 crop season, the lowest precipitation amounts were recorded in November and December, close to 0 mm at the end of full bloom and grape ripening (Figure 1a–c). Average air temperatures were the highest in December and January in all crop seasons (close to 22 °C), which is the period when the berries are ripening and the grapes are being harvested. In the 2018/19 crop season, precipitation volumes were higher than the climatological normal observed over the previous 17 years (Figure 1d). However, in 2020, precipitation throughout the year was around 550 mm, which was below the average for 2018 and 2019. All three crop seasons had average temperatures lower than the climatological normal observed over the previous 17 years.

### 2.1. P Concentration in Leaves and Grape Production

**Figure 2:**
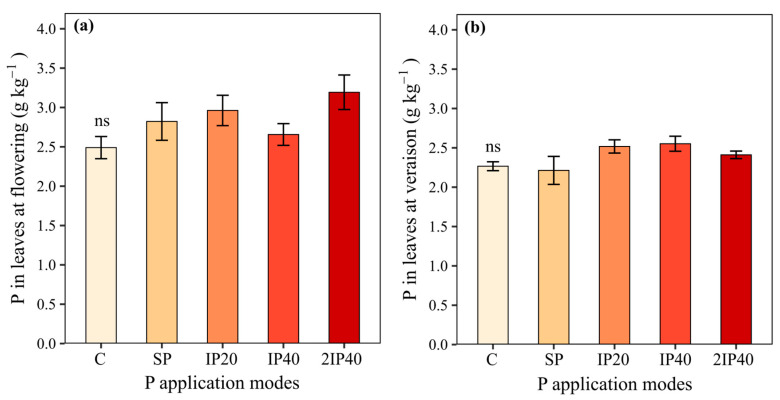
P concentrations in leaves at flowering (**a**) and at *veraison* (**b**) evaluated over three crop seasons of ‘Pinot Noir’ grapevines subjected to P application modes to a Typic Hapludalf soil in from southern Brazil. ns = non-significant difference by Tukey’s test (*p* < 0.05). Without P application (C), P on the soil surface without incorporation (SP), P incorporated at 20 cm (IP20), P incorporated at 40 cm (IP40), and twice P dose incorporated at 40 cm (2IP40).

**Figure 3:**
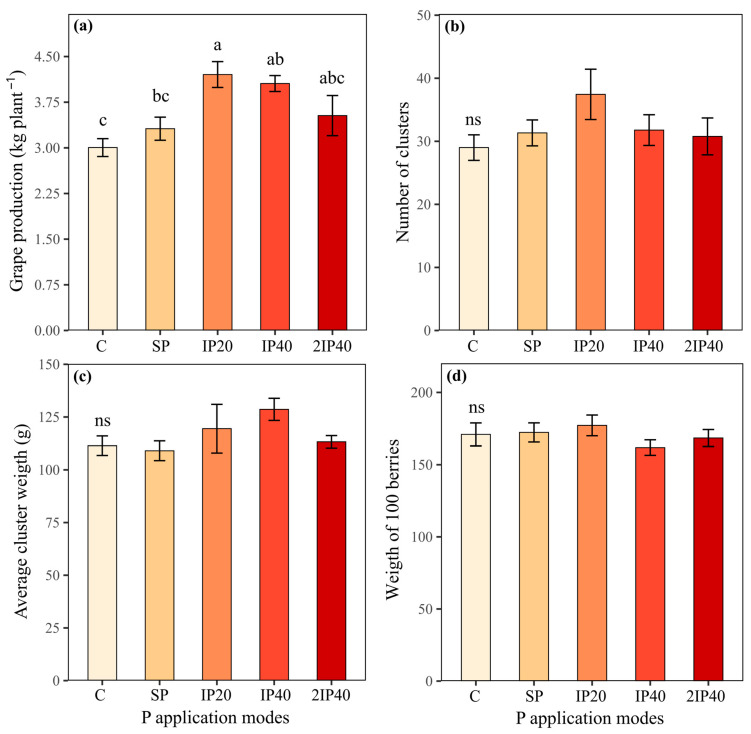
Grape production per plant (**a**), number of clusters (**b**), average weight of clusters (**c**) and weight of 100 berries (**d**) evaluated over three crop seasons of ‘Pinot Noir’ grapevines subjected to P application modes in a Typic Hapludalf soil from southern Brazil. Lowercase letters compare the means of the treatments (P application modes) by Tukey’s test (*p* < 0.05). ns = non-significant difference. Without P application (C), P on the soil surface without incorporation (SP), P incorporated at 20 cm (IP20), P incorporated at 40 cm (IP40), and twice P dose incorporated at 40 cm (2IP40).

The P concentrations in the leaves collected at full bloom and *veraison* did not differ between the treatments (C, SP, IP20, IP40, and 2IP40) (Figure 2a,b). The highest grape production was observed in the vines grown in the soil with IP20 (Figure 3a). The grape production obtained by IP20 did not differ statistically from IP40, which was equal to the production observed in 2IP40 (Figure 3a). The number of clusters per plant, the average weight of clusters and the weight of 100 berries did not differ statistically between the P application modes (Figure 3b,d). However, the highest values were observed in the soil subjected to IP20 application (Figure 3b,d).

### 2.2. Composition of the Grape Must

**Figure 4:**
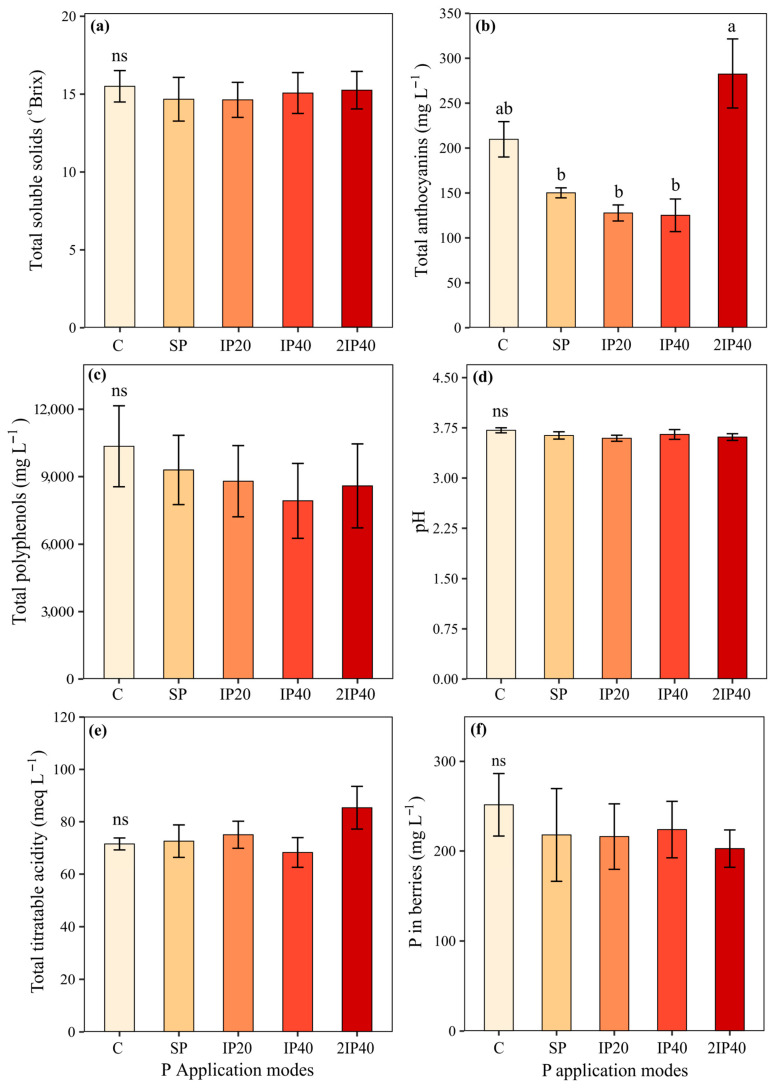
Total soluble solids (**a**), total anthocyanins (**b**), total polyphenols (**c**), pH (**d**), total titratable acidity (**e**), and P concentration in the grape must (**f**) evaluated over three crop seasons of ‘Pinot Noir’ grapevines subjected to P application modes in a Typic Hapludalf soil from southern Brazil. Lowercase letters compare the means of the treatments (modes of P application) using Tukey’s test (*p* < 0.05). ns = non-significant difference. Without P application (C), P on the soil surface without incorporation (SP), P incorporated at 20 cm (IP20), P incorporated at 40 cm (IP40), and twice P dose incorporated at 40 cm (2IP40).

The highest concentrations of TA were observed in the grape must of grapevines grown in soil with 2IP40 and C (Figure 4b). The values of TSS, TP, pH, TTA and P concentration in the grape must did not differ statistically between the P application modes (Figure 4a,c–f).

### 2.3. Phosphorus Content in the Soil Profile

**Figure 5:**
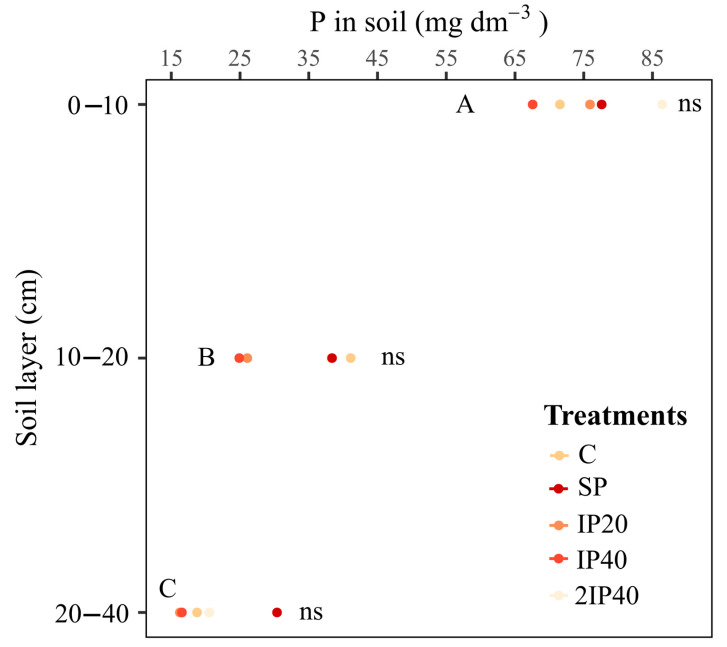
P content in the soil extracted by Mehlich-1 in the 0–10, 10–20 and 20–40 cm layers, in a vineyard evaluated over three crop seasons subjected to P application modes to a Typic Hapludalf soil from southern Brazil. Capital letters compare the P content between soil layers using Tukey’s test (*p* < 0.05). ns = non-significant difference between treatments in the same soil layer. In the 2018/19 crop season, the P content in the 20–40 cm layer was not determined. Without P application (C), P on the soil surface without incorporation (SP), P incorporated at 20 cm (IP20), P incorporated at 40 cm (IP40), and twice P dose incorporated at 40 cm (2IP40).

The contents of available P did not differ statistically between the P application modes in the 0–10, 10–20 and 20–40 cm layers (Figure 5). However, in the 0–10 cm layer, the P contents were higher in all P application modes compared to the values observed in the 10–20 and 20–40 cm layers (Figure 5). It should be noted that P contents in the 0–10 cm layer tended to be higher in the soil in the 2IP40 treatment (Figure 5). Considering the crop seasons effect, individually, the results show that the highest P contents in the 0–10 and 10–20 cm layers are observed in the 2018/19 and 2020/21 crop seasons in the 2IP40 treatment. In the 2019/20 crop season, the highest P contents are observed in the SP treatment in the three soil layers (Supplementary Material Table S1).

### 2.4. Variance Components and Inference Tree

**Figure 6:**
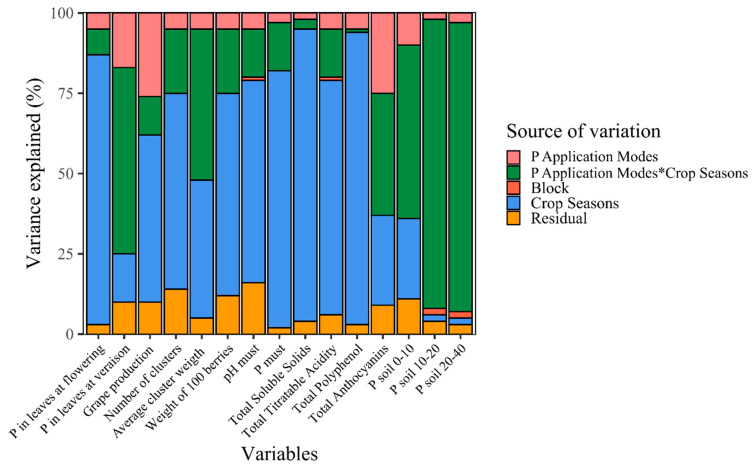
Proportion of variance explained by each source of variation for each response variable. The colors represent the source of variation (P application modes, crop seasons, blocks, residuals, and interaction between P application modes and crop seasons).

In the variance components analysis, the crop season (year) effect explained a large part of the variation in most of the variables, especially the variables of P concentration in the leaves at flowering, grape production, number of clusters per plant, weight of 100 berries, and chemical parameters of the grape must (Figure 6). The interaction between the P application modes and crop season explained more than 90% of the variation in the P content in the soil (10–20 and 20–40 cm layer) and around 50% of the variation in P content in the 0–10 cm layer, P in the leaf at *veraison* and average cluster weight (Figure 6). The P application modes in isolation played a minor role in explaining the total variation in the variables (around 10%), showing the greatest response in the P concentration in leaves collected at *veraison* and approximately 25% in production per plant and TA (Figure 6). 

**Figure 7:**
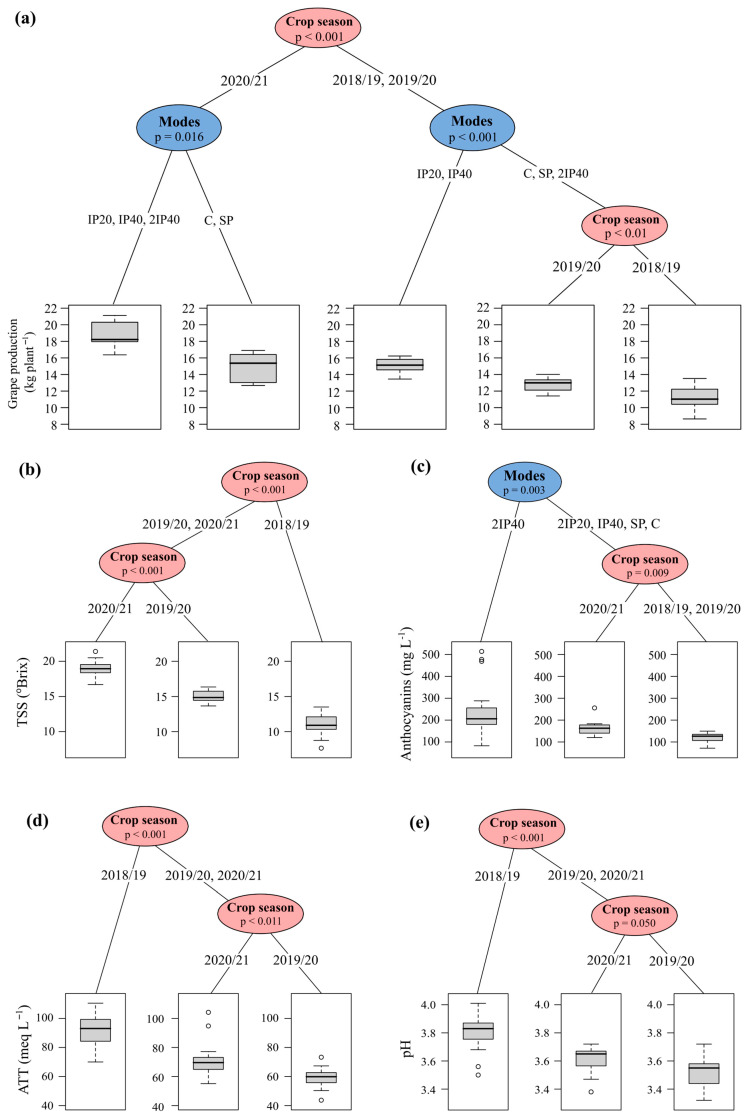
Conditional inference tree showing the effect of P application modes and crop seasons on grape production (**a**): total soluble solids—TSS (**b**), total anthocyanins—TA (**c**), total titratable acidity—TTA (**d**) and pH of the grape must (**e**).

The inference tree separated the crop seasons into two groups. In the 2020/21 crop season, the soils subjected to the application of treatments with incorporated P had higher productivity (Figure 7a). The second group, made up of the first two crop seasons, separated the IP20 and IP40 soils, which achieved higher productivity compared to the other P application modes (Figure 7a). The crop season factor had the greatest effect in conditioning the separation of the groups of values for the grape must composition parameters TSS, TTA and pH (Figure 7b,d,e), with the highest TSS values being observed in the 2020/21 crop season, while the highest TTA and pH values were observed in the 2018/19 crop season. On the other hand, the P application mode factor had a greater effect on grouping TA values (Figure 7c) with the highest values being observed in the 2IP40 application mode.

### 2.5. Principal Component Analysis

**Figure 8:**
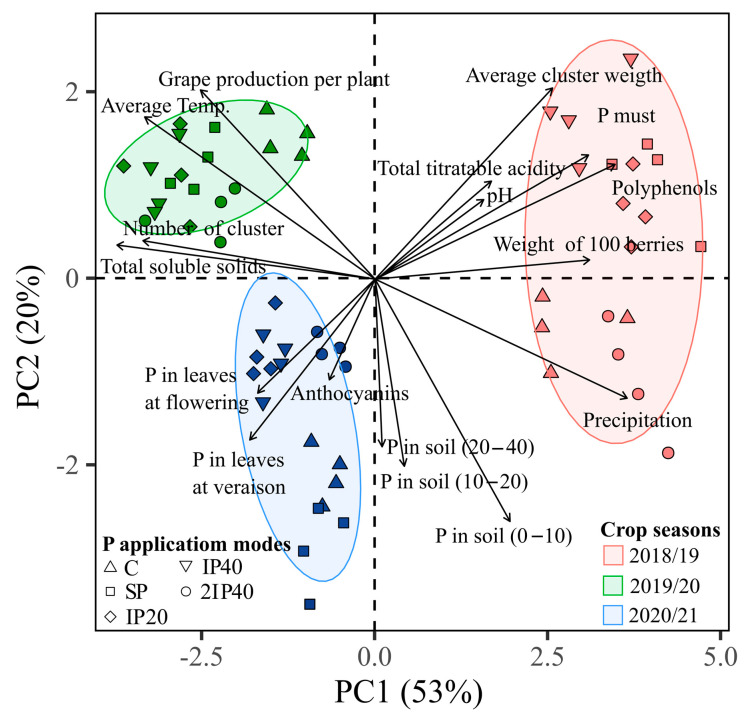
Relation between principal component 1 (PC1) and principal component 2 (PC2), for P concentration in the soil and leaves, grape production and its components, grape must parameters and climatic variables evaluated over three crop seasons to P application modes in the soil. Without P application (C), P on the soil surface without incorporation (SP), P incorporated at 20 cm (IP20), P incorporated at 40 cm (IP40), and twice P dose incorporated at 40 cm (2IP40).

The principal component analysis (PCA) was carried out by extracting only the first two components, since the sum of the PC1 (53%) and PC2 (20%) components explained 73% of the variance in the original data (Figure 8). PC1 was mainly influenced by precipitation, average cluster weight, TP, TTA, pH and P in the grape must (Figure 8). These variables showed an inverse correlation with precipitation and P concentration in leaves and soil. The PC2 was greatly influenced by the variables production, number of clusters, TSS and TA, which showed a positive correlation with the P concentration in the leaves and the average air temperature (Figure 8). Overall, the PC1 and PC2 scores showed no effect of the treatments; however, there was a strong effect of the crop season factor on the variance of the data, with the PC scores separating the samples from the three crop seasons evaluated. Thus, the positive group (to the right of the X axis) is composed of observations from the 2018/19 crop season, which is associated with a lower relative production compared to the other crop seasons. The negative group (to the left of the X axis) was composed of observations from the 2019/20 and 2020/21 crop seasons.

## 3. Discussion

### 3.1. Phosphorus Concentration in Leaves and Soil

The P concentrations at flowering and *veraison* did not differ between P application modes (Figure 2). This may be justified because grapevines have forms of P accumulated in perennial organs, such as roots, stems, and branches of more than one year, which can be redistributed to growing annual organs, such as leaves and clusters [7,8,9]. Normally, in fruit trees, P reserves in perennial organs can decrease significantly or even be depleted over three years. This is because perennial crops in subtropical climates, such as grapevines, have great potential for biennial bearing, transferring their nutrient reserves to the growth and vegetative structure for one or two years, thus providing the necessary support for long-term fruiting [10]. When this happens, the fruit tree begins to absorb greater quantities of the nutrients derived from the soil [7]. However, this may not have been observed because the grape productivity was not very high (on average 15 t ha^−1^), while the dose of P applied in the vineyard considered an expected grape productivity of 25 t ha^−1^. Another explanation is that the vines reached the luxury consumption of P, i.e., the absorption of P that does not lead to an increase in the P concentrations in grapevines [11].

The foliar P concentrations at flowering were higher than those observed at *veraison*. This is probably due to the dilution effect that occurs with leaf growth and the redistribution of nutrients to other plant organs at the end of the cycle [12,13,14]. In addition, P forms can be redistributed from leaves to organs undergoing intense cell division and elongation, which causes an increase in dry matter, such as clusters [15]. On the other hand, the P concentrations in the berries tended to be higher in the grapevines without P application, which could be explained by the lower grape production, which would increase the concentration of P in the tissue (concentration effect). This may be because in the three crop seasons of evaluation, precipitation was lower than climatological normal, even more so in the 2020/21 crop season, and this may have been decisive for our results. Since, it rained less, the grapevines produced less than expected and did not respond sufficiently to treatments. Moreover, there is a gradient of P content in the soil, with higher P content in the superficial layers of the soil, decreasing significantly in depth. This is due to the fact that much of the P applied is rapidly adsorbed to the functional groups of inorganic reactive particles, which reduces its mobility in the soil [16]. Another reason is due to the cycling and deposition of P on the soil surface by the biomass of cover crops and leaves and branches of grapevines. Furthermore, the application of twice the recommended dose of P did not increase P values at depth. This may be related to greater exposure of P to adsorption sites and the dilution effect of P with soil disturbance. Another explanation for this would be because the P content in the experiment installation (16.1 mg kg^−1^) is considered low in soils with clay less than 20%, according to [17]. Also, due to the high sand content in the experiment soil, the P can be absorbed by cover plants, contributing to the increase in organic P fractions. This increases P accumulation on the soil surface.

### 3.2. Grape Production and Chemical Parameters of the Grape Must

The highest grape production was observed in the grapevines grown in the soil in which the fertilizer was applied and incorporated with a subsoiler, up to 20 cm (IP20) (Figure 3a). Since we do not observe differences in the P content between treatments, we can infer that the production increases were related to soil mobilization, but not by P rates or by P incorporation, in the first two crop seasons (Supplementary Material Table S1). The soil mobilization by the subsoiler does not sufficiently incorporate P into deeper soil layers.

The lowest grape production (Figure 3a) was observed in grapevines grown in soil without P application (control), which is followed by plants subjected to P application on the soil surface (SP). This shows that grapevines grown in sandy soils increase grape production when subjected to P application. The justification is that sandy soils have a lower P adsorption capacity and, as a result, part of the P applied to the soil can increase the more labile P forms, which can be absorbed by the vines [7,16]. On the other hand, applying P to the soil surface does not seem to be a suitable strategy for increasing the productivity of grapevines, which is probably because P is adsorbed to the functional groups on the surface of inorganic particles and, as a result, its migration is very slow or practically does not occur in a short space of time [18], even in sandy soil [19]. As a result, the amount of P absorbed tends to decrease over the years, increasing the potential for increased productivity. In addition to the effect on nutrient availability, subsoiling can mitigate the effects of soil compaction, reducing soil density and increasing soil porosity [20]. This can contribute to greater exploitation of the grapevine root system, increasing the absorption of water and nutrients, which can increase the grapevine production. This is because along the vine row, the soil is deeply ripped, which facilitates better root penetration into the subsoils and enhanced surface layer water drainage.

The P application modes did not affect the values of TSS, pH, TP and TTA of the grape must. However, the highest values of TA were observed in the grapevines subjected to the application of twice the dose of P, which was incorporated up to 40 cm (2IP40) (Figure 4b and Figure 7c). This may probably have been due to the increase in P content in the soil, which increases its absorption by the grapevines. On the other hand, high values of TA were also observed in the vines grown in soil without P application (control). This may have occurred because this treatment showed lower grape production, which indicates the effect of P concentration in the berry. On the other hand, grapevines grown in soils without phosphate fertilization may show less vigor in the shoot. When this happens, anthocyanins are less likely to be redistributed from the clusters to growing annual organs such as leaves and branches [8,14]. In addition, soils without phosphate fertilization or with low P concentrations stimulate the synthesis of anthocyanins [21,22].

From the PCA, we can see the most pronounced crop season effect (Figure 8), associated with the oscillation in grapevine production between the years, which is related to the climatic variation between the three crop seasons evaluated (Figure 1b). This is natural in perennial plants, which have their photoassimilate reserves redistributed with each new production cycle based on remobilization from source organs to drain organs [7,23,24]. In addition, the climatic variables temperature and precipitation, the P application modes, 2IP40, and the P concentration in leaves and soil were correlated with the highest production of grapes and anthocyanins in the must, especially in the 2020/21 crop season (Figure 8), which is characterized as a hot and dry crop season in the critical months (November and December) for grape ripening in the study region (Figure 1c). Therefore, the need to seek more precise technical phosphate fertilization recommendations for fruit trees is evident, especially considering the climate change scenario which can interfere on grape productivity and must composition in vineyards.

## 4. Materials and Methods

### 4.1. Experimental Layout and Treatments

The study was carried out in a vineyard in Santana do Livramento city, Rio Grande do Sul State (Latitude 30°48′58″ S; Longitude 55°26′40″ W, and at 208 m of altitude), which is located in the Campanha Gaúcha region, southern Brazil (Supplementary Material Figure S1). The region’s climate was classified as humid subtropical (Cfa), according to the Köppen and Geiger classification [25]. The average annual temperature is 18.4 °C, and the average annual precipitation is 1532 mm. The average temperature and precipitation data during the study period, the climatological normal and the historical climate are presented in Figure 1.

The soil in the experiment is classified as arenic dystrophic red Argisol [26], corresponding to Typic Hapludalf [27]. Before the experimental unit was set up, the soil had the following physical and chemical attributes in the 0–20 cm layer: 775.7, 105.7, and 118.5 g kg^−1^ of sand, silt, and clay, respectively (pipette method); pH units in water = 7.2 (1:1 ratio); 0.4, 2.2 and 1.2 cmolc kg^−1^ of exchangeable aluminum (Al), magnesium (Mg) and calcium (Ca) (KCl 1 mol L^−1^), respectively; 16.1 and 57.7 mg kg^−1^ of available phosphorus (P) and potassium (K) (Mehlich-1), respectively. 

The vineyard was planted in 2010 in a natural field. Pre-planting fertilizer was applied with 45 kg K_2_O ha^−1^ (potassium chloride—58% K_2_O) and 45 kg P_2_O_5_ ha^−1^ (triple superphosphate—46% P_2_O_5_). The fertilizers were applied launched in total area, on the soil surface, which was followed by incorporation of up to 20 cm, with plowing, and harrowing [28]. The ‘Pinot Noir’ cultivar (*Vitis vinifera* L.) was grown in an espalier system with a spacing of 2.5 m between rows and 1.0 m between plants (density of 4000 plants ha^−1^).

The treatments were applied in October 2018 with the grapevines being subjected to a control treatment (C—without P application); P on the soil surface without incorporation (SP), P incorporated at 20 cm (IP20), P incorporated at 40 cm (IP40), and twice P dose incorporated at 40 cm (2IP40). The doses of P were set according to the recommendation proposed by [17], which takes into account the content of available P in the soil (Mehlich-1), the clay content of the soil, and the expected grape production. The leaf concentration was considered to be in the normal range [17]. During the three crop seasons evaluated, the experimental area had annual precipitation below normal for the region. The treatments were applied only at the time of the study implementation (2018), and the evaluations were carried out in the 2018/19, 2019/20 and 2020/21 crop seasons.

Thus, at the time the experiment was set up, in the diagnostic layer (0–20 cm), the soil had 16 mg dm^−3^ of available P (Mehlich-1), which is considered low for these types of soils, in the southern region of Brazil, and 118.50 g kg^−1^ of clay (pipette method), and grape production was expected to be greater than 25 t ha^−1^. Therefore, the dose applied was 80 kg P_2_O_5_ ha^−1^ (triple superphosphate—46% P_2_O_5_). In the SP treatment, the P was applied to the surface of the soil without incorporation. In the IP20, IP40, and 2IP40 treatments, the P was applied to the soil surface and incorporated using a subsoiler (model ASGFPA- Industrial BECKER), with five rods attached to a tractor, with 75 horsepower (hp) with a cutting width of 1.50 m and a height of 0.61 cm, at a distance of 70 cm from the stem of the vines. The application of the P dose as well as the incorporation of P in the treatments were carried out only in the year of implementation of the experiment. Over the course of the experiment, 45 kg N ha^−1^ (whose source was urea, 45% N) and 45 kg K_2_O ha^−1^ (whose source was potassium chloride—KCl, 58% K_2_O) were applied annually (2018, 2019 and 2020). The urea and KCl were distributed on the soil surface, without incorporation, in the region of the canopy projection, after the grapevines had started to budbreak.

The experimental design was randomized blocks with four replications. Each replication had 15 plants at a distance of 1.0 m along the rows and 2.5 m between the rows (4000 vines ha^−1^); and the 10 central plants were evaluated. *Paspalum notatum*, *Paspalum plicatulum*, *Desmodium affine* Schltdl, *Lolium multiflorum*, *Vicia sativa* and *Raphanus raphanistrum* predominated in the vineyards inter-rows. The shoot of the cover plants was cut at a height of approximately 10 cm, three times during the grapevine cycle, and deposited on the soil surface. In the rows where the grapevines were planted, the cover plants were desiccated with a non-residual herbicide three times a year. Each year, the vines underwent winter pruning, leaving 12 buds per branch, to regulate crop level and achieve the desired grape must composition. The vines were not subjected to irrigation systems.

### 4.2. Leaf Sampling and P Determination

In 2018/19, 2019/20, and 2020/21 crop seasons, at full bloom and *veraison* (when the grapes changed color), complete leaves (leaf + petiole) were collected opposite the clusters in the middle third of the branch of the year. The leaves were dried in an oven with forced air circulation at 65 °C until they reached a constant mass, ground in a Willey mill and passed through a sieve with a mesh size of 2 mm. The leaves were prepared and then subjected to digestion with nitroperchloric acid (HNO_3_:HClO_4_—3:1 ratio *v*/*v*) [29]. The concentration of total P in leaves was then determined using a UV-Visible spectrophotometer (Bell Photonics, 1105, São Paulo, Brazil), at 882 nm, as proposed by [30]. 

### 4.3. Grape Production and Its Parameters

When the grapes were harvested (January 2019, 2020 and 2021), the clusters from each plant were picked separately, counted and weighed using a three-digit precision digital scale (Walmur, 50K, Rio Grande do Sul, Brazil) to obtain the production per plant. Subsequently, two clusters per repetition were set aside. Berries from the upper, middle and lower thirds of the selected clusters were collected and weighed (Bel engineering, L303i, Monza, Italy) to determine the weight of 100 berries. The berries were set aside for later chemical analysis of the grape must composition. The average cluster weight was calculated by dividing the total production by the number of clusters per plant.

### 4.4. Chemical Analysis of the Grape Must

The berries were manually peeled, separating the seed and pulp from the skin. The pulp and seed (must) were homogenized. The must was then digested in sulfuric acid. To achieve this, 2 mL samples of must were added to 50 mL glass digestion tubes and treated with an acid solution (H_2_SO_4_:H_2_O_2_—2:1 ratio *v*/*v*). After digestion, the samples were cooled under laboratory conditions to room temperature (22 °C). After cooling, the samples were diluted with distilled water (extraction dilution). The total P was determined using a UV-visible spectrophotometer (Bell Photonics, 1105, São Paulo, Brazil) at 882 nm, as proposed by [30]. The total soluble solids (TSSs) in the must were determined by direct refractometry (Reichert Technologies, Brix/RI-Chek, New York, United States), according to [31]. The results were expressed in °Brix. The pH of the grape must was determined using a digital bench pH meter with automatic temperature control (Digimed, DM-22, São Paulo, Brazil), using 4.0 and 7.0 buffer solutions. Total titratable acidity (TTA) was determined by titrating 10 mL of must with 0.1 N NaOH and phenolphthalein (1%) as an indicator. The results were expressed in meq L^−1^.

Total anthocyanins (TAs) and total polyphenols (TPs) were determined in the grape skins. The skin was crushed with a solution of ethanol (70:30) acidified (1% HCl) in an attached mixer in the ratio (3:1—ratio *v*/*m*). The extract was kept in a beaker away from light for 30 min at room temperature (20 °C ± 1 °C). It was then centrifuged at 3500 RPM for five minutes. The supernatant was stored in a refrigerator for analysis. The TA was determined using the pH difference method [32], in which it is diluted in two buffer systems: potassium chloride pH 1.0 (0.025 mol L^−1^) and sodium acetate pH 4.5 (0.4 mol L^−1^). TP was determined according to the Folin–Ciocalteau colorimetric method, described by [33], with adaptations. To achieve this, 50 µL of the extracted sample was added to test tubes and mixed with 450 µL of 1% acidified extractant solution. Subsequently, 200 µL of the extract obtained was again added to test tubes, and then 1000 µL of diluted Folin’s reagent (1:10) was added; after 3–8 min, 800 µL of 7% Na_2_CO_3_ was added. The samples were left to stand in the dark and after 2 h were read in a UV-visible spectrophotometer (Bell Photonics, 1105, São Paulo, Brazil) at 765 nm. The TP was determined using a calibration curve with gallic acid (0–100 mg L^−1^), and the results were expressed in mg L^−1^.

### 4.5. Soil Sampling and P Analysis

In the 2018/19, 2019/20 and 2020/21 crop seasons, the soil was collected in March, always after the grape harvest, in the 0–10, 10–20 and 20–40 cm layers, between the rows of the vineyard. The soil was air-dried, passed through a 2 mm mesh sieve and set aside. Available P was extracted using a Mehlich-1 extractor and determined using a UV-visible spectrophotometer (Bell Photonics, 1105, São Paulo, Brazil) at 882 nm, according to the methodology proposed by [30].

### 4.6. Statistical Analysis

All the study variables were subjected to variance component analysis (‘VCA’ package [34]), quantifying the contribution of the following sources of variation: treatments (C, SP, IP20, IP40 and 2IP40), crop seasons (2018/19, 2019/20 and 2020/21), blocks, residues and the interaction between treatments and crop seasons. Next, the variables grape production and must composition parameters were subjected to conditional inference tree analysis (‘party’ package [34]) with the aim of identifying the effect of phosphorus application methods (treatments) and crop seasons. After that, all the study variables were subjected to analysis of variance (ANOVA) using the R package ‘ExpDes.pt’ [34], considering the ‘treatments’ factor as a fixed effect and the blocks as a random effect. The normality of the residuals was tested using the Shapiro–Wilk test to see if any transformation was necessary. Whenever the null hypothesis (equal means) was rejected with an alpha equal to 0.05, the means were compared using Tukey’s test (p < 0.05). Finally, all the data were subjected to principal component analysis (PCA) to explore the variance of the data, allowing for the identification of more complex interactions between the variables, as well as verifying the similarity/dissimilarity between the crop seasons. PCA was carried out using the R packages ‘FactoMineR’ and ‘factoextra’ [34].

## 5. Conclusions

The application of the recommended dose of P on the soil surface or incorporated up to 20 and 40 cm, as well as the application of twice the recommended dose of P and incorporated up to 40 cm, did not affect the P concentrations in leaves, berries or the grape must composition. However, it is recommended to consider applying the treatments in periods with less rainfall. This is because, in crop seasons (year: climate variables) with less rainfall, an increase in grape production and anthocyanin concentration in the grape must of ‘Pinot Noir’ vines grown in subtropical regions was observed especially when P was incorporated up to the 20 and 40 cm layers.

## Data Availability

The datasets generated during the current study are available from the corresponding author on reasonable request. The data are not publicly available due to [being part of a higher research project that has been conducted].

## Supplementary Materials

The following supporting information can be downloaded at: https://www.mdpi.com/article/10.3390/plants13172434/s1, Table S1: P content in the soil extracted by Mehlich-1 in the 0–10, 10–20 and 20–40 cm layers, in a vineyard subjected to P application modes to the soil over three crop seasons in an *Typic Hapludalf* from southern Brazil. Figure S1. Location of the experimental area.
