# Supplementary material for: Strategies to Increase the Phosphorus Content in the Soil Profile of Vineyards Grown in Subtropical Climates
Source: Plants (Basel). 2024 Aug 31;13(17):2434. doi: 10.3390/plants13172434 (PMC11397632; doi:10.3390/plants13172434)
Supplement: Supplementary file 1 [file plants-13-02434-s001.zip › plants-3102572-supplementary/plants-3102572-supplementary S2.pdf]

Table S1. P content in the soil extracted by Mehlich-1 in the 0-10, 10-20 and 20-40 cm layers, in a vineyard subjected to P application modes to the soil over three crop seasons in an *Typic Hapludalf* from southern Brazil.

Crop seasons	Layer (cm)	Treatments				
		**C**	**SP**	**IP20**	**IP40**	**2IP40**
2018-19	0-10	69.19 Ac (± 4.37)	78.16 Abc (± 5.49)	75.20 Abc (± 5.32)	89.46 Ab (± 5.77)	125.97 Aa (± 7.55)
	10-20	33.62 Bb (± 0.84)	31.27 Bb (± 5.61)	40.12 Bb (± 7.07)	10.83B c (± 4.01)	52.17 Ba (± 3.09)
2019-20	0-10	107.60 Ac (± 6.15)	102.77 Aab (± 10.50)	63.01 Ac (± 8.10)	81.00 Abc (± 13.88)	66.55 Ac (± 9.29)
	10-20	52.18 Bb (± 6.17)	66.98 Ba (± 3.51)	14.58 Bd (± 7.63)	30.50 Bc (± 4.15)	9.29 Bd (± 0.30)
	20-40	24.01 Cb (± 1.51)	40.77 Ca (± 2.53)	14.75 Bbc (± 8.86)	22.34 Bb (±8.62)	9.26 Bc (± 1.03)
2020-21	0-10	43.82 Ac (± 7.43)	50.77 Acd (± 4.77)	67.53 Aab (± 2.26)	61.00 Abc (± 2.22)	71.52 Aa (± 2.20)
	10-20	32.92 Bb (± 8.06)	19.64 Bb (± 7.24)	32.98 Bb (± 4.70)	31.39 Bb (± 0.39)	59.63 Ba (± 10.92)
	20-40	8.37 Cd (± 3.20)	7.98 Cd (± 0.94)	25.24 Cb (± 6.33)	17.03 Cc (± 3.15)	41.52 Ca (± 1.69)

Values in parentheses represent the standard deviation. Lowercase letters compared the P content of each soil layer between treatments and uppercase letters compared each treatment between soil layers by Tukey's test ($p < 0.05$). In the 2018/19 crop season, the P content in the 20-40 cm layer was not determined. Without P application (C), P on the soil surface without incorporation (SP), P incorporated at 20 cm (IP20), P incorporated at 40 cm (IP40), and twice P dose incorporated at 40 cm (2IP40).
