# Supplementary figures and images for: Strategies to Increase the Phosphorus Content in the Soil Profile of Vineyards Grown in Subtropical Climates
Source: Plants (Basel). 2024 Aug 31;13(17):2434. doi: 10.3390/plants13172434 (PMC11397632; doi:10.3390/plants13172434)

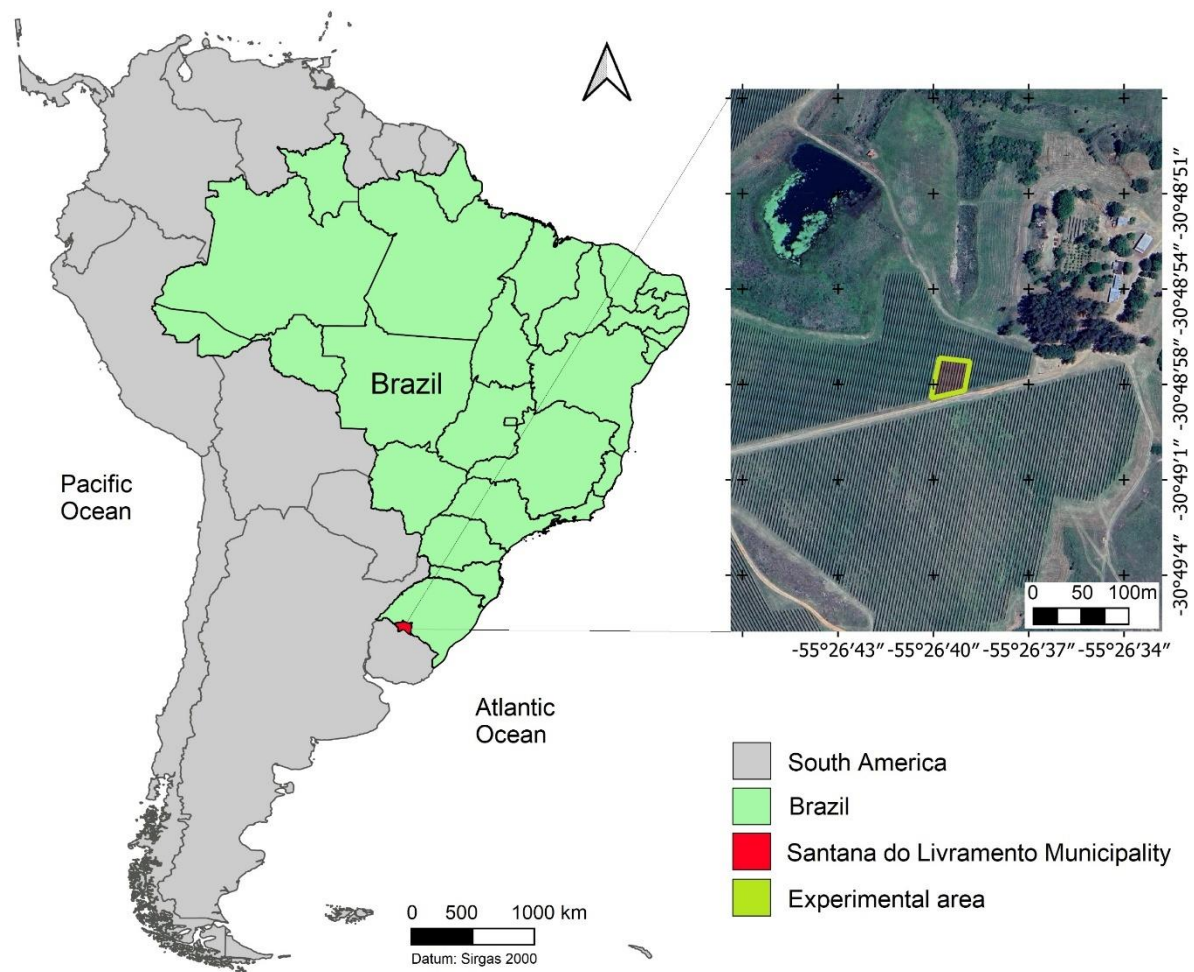

**Supplementary material Figure S1.** Location of the experimental area.

Supplement: Supplementary file 1 [file plants-13-02434-s001.zip › plants-3102572-supplementary/plants-3102572-supplementary S1.pdf]
